# Microencapsulation of Bioactive Principles with an Airless Spray-Gun Suitable for Processing High Viscous Solutions

**DOI:** 10.3390/jfb4040312

**Published:** 2013-11-19

**Authors:** Moreno Cocchietto, Paolo Blasi, Romano Lapasin, Chiara Moro, Davide Gallo, Gianni Sava

**Affiliations:** 1 Callerio Foundation Onlus, Institutes of Biological Researches, Trieste 34127, Italy; E-Mails: chiaramoro87@gmail.com (C.M.); g.sava@callerio.org (G.S.); 2 Department of Pharmaceutical Sciences, University of Perugia, Perugia 06123, Italy; 3 Department of Materials and Natural Resources, University of Trieste, Trieste 34127, Italy; E-Mail: romano.lapasin@di3.units.it; 4 Department of Pharmaceutical and Pharmacological Sciences, University of Padua, Padua 35131, Italy; E-Mail: davide.gallo.3@studenti.unipd.it; 5 Department of Life Sciences, University of Trieste, Trieste 34127, Italy

**Keywords:** microencapsulation, airless spray-gun, lysozyme, alginate-chitosan microparticles

## Abstract

Purpose: to design, assemble and test a prototype of a novel production plant, suitable for producing microparticles (MPs) by processing highly viscous feed solutions (FSs). Methods: the prototype has been built using a commercial air compressor, a piston pump, an airless spray-gun, a customized air-treatment section, a timer, a rotating base, and a filtration section. Preliminary prototype parameter setting was carried out to individuate the best performing nozzle’s dimension, the nebulization timing, and the CaCl_2_ concentration in the gelation fluid. In addition, prototype throughput (1 L to 5 L) and the range of practicable feed solution (FS) viscosities were assayed. A set of four batches was prepared in order to characterize the MPs, in terms of mean particle size and distribution, flow properties, swelling, encapsulation efficiency and release. Results: according to a qualitative scoring, the large nozzle was suitable to nebulize FSs at a higher alginate concentration. Conversely, the small nozzle performed better in the processing of FSs with an alginate concentration up to 2% w/v. Only at the highest degree of viscosity, corresponding to 5% w/v of alginate, the FS processing was not technically possible. Among the CaCl_2_ concentrations considered, 15% w/v was recognized as the most versatile. The prototype appears to be convenient and suitable to grant a high yield starting from 2 L of FS. The flow behavior of the FSs assayed can be satisfactorily described with the Carreau-Yasuda equation and the throughput begins to slightly decrease for FSs at alginate concentrations exceeding 3% w/v. MP morphology was irregular with crumpled shape. The angle of repose indicates a good flowability and the release studies showed gastro-resistance and potential prolonged release applications. Conclusions: the novel prototype of production plant is suitable to process large amounts (2 L or more) of FSs, characterized by a high viscosity, to produce MPs suitable for bioactive principle delivery.

## 1. Introduction

Microencapsulation has a rising importance for pharmaceutical, biomedical, cosmetic, zoo-technical and food-related fields [1]. A number of techniques and technologies are being used to produce systems on the micro-scale [2,3]. The available preparation methods and technologies suffer from different limitations, such as a partial reliability, difficulties in the scale-up for industrial manufacturing, cost-effectiveness of the process and limited materials suitable for processing. In fact, only few technologies, such as spray-drying and fluid bed, are largely applied on an industrial scale.

In a previous work, a versatile lysozyme (LZ)-containing microparticulate system, suitable for the oral delivery of different bioactive principles and vaccines, was developed [4,5]. Alginate, a natural polymer extracted from seaweed, approved for food, cosmetic, and pharmaceutical uses and suitable for a broad range of applications, was chosen as wall material [6,7,8]. The beads obtained were matrix-type capsules: they hold any filling throughout the bead rather than having a distinct shell as in core-shell capsule types. The production method relied on alginate ionotropic gelation starting from w/o/w emulsions.

LZ-containing oral administrable microsystems (MSs), prepared as described above, were used for the chemoprevention of the diabetic nephropathy [9,10]. Loaded with inactivated *Photobacterium damsela subsp. piscicida* or with bacterin^®^, the same MS was employed for the oral vaccination in aquaculture against fish photobacteriosis [11] and fish lactococcosis, respectively.

After the promising results obtained with the above mentioned microparticulate systems on a diabetes-induced preclinical model and on a small-scale vaccination trial on fishes, we envisaged the necessity of a larger scale production system in order to perform further studies.

Preparation techniques such as emulsification, laboratory-scale spray-drying and coaxial air driven technology presented a number of issues mainly related to the difficulty to obtain a large amount of product (in the range of 100 g/die) with the requested characteristics. Furthermore, the cost-effective food-grade alginates available on the market were highly viscous at the suitable concentrations, making unusable the prototype based on coaxial air-driven technology and preventing any attempt to use spray-drying technology.

In order to make possible the processing of highly viscous biopolymer solutions to produce MPs and to increase the daily productivity at reasonable costs, an original prototype, based on airless spray-gun technology was designed, developed, assembled and validated [12].

In this study, the prototype is described and its efficiency, mainly focusing on the relation among FS viscosity and throughput, was evaluated. Besides, a series of microparticle (MP) batches was produced and four of them were fully characterized in terms of mean particle size and distribution, morphology, angle of repose, swelling, loading, and release.

## 2. Results and Discussion

### 2.1. Airless Spray-Gun Description

**Figure 1:**
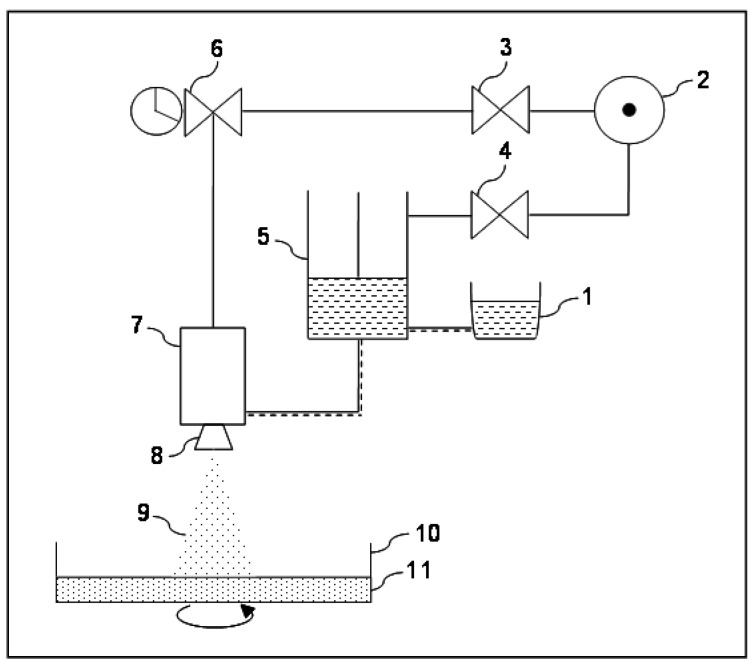
Schematic view of the prototype. Description: 1. reservoir for feed solution; 2. pressurized air source with drying unit and filtering station; 3–4. pneumatic circuit pressure regulators; 5. piston pump; 6. nebulization duration and frequency controlling device; 7. air-less nebulizing unit; 8. tip with small hole; 9. nebulized feed solution; 10. collecting tank (placed over an adjustable speed rotating platform); and 11. gelation and coating fluid.

The microencapsulation instrument hereafter described was able to manufacture large MP batches by processing high viscous polysaccharide solutions. The airless spray-gun can be considered a hybrid system in between a spray-dryer and a microencapsulation instrument for polymer ionotropic gelation after dropping [12]. In particular, the viscous FS containing the active ingredient is nebulized by an airless spray-gun in a rotating collecting tank containing the gelation solution. Particles are then recovered simply by filtration, washed, and dried (Figure 1) [12].

Briefly, the air, generated by the compressor, is firstly treated to remove humidity and impurities using an air dryer and a battery of filters to remove dust and the microscopic droplets of oil that can be generated by the air compressor. The last filter of the battery contains active carbon. Even though the air does not enter in contact with the feed solution or with the product during manufacturing, air filtration is aimed to prolong the life span of the circuits and the components [13]. After treatment, the air is split into two circuits. The first leading to a piston pump (used to pump under high pressure the FS to the airless spray-gun) where the pressure is increased by 15 fold and, the second, operating at lower pressure, connected to a timer regulating the duration of the single atomization impulse and the pauses between two consecutive impulses. Downstream to the timer, the air circuit is connected to the spray-gun where it regulates the opening and the closing of the valve controlling the FS flow. FS is forced through a nozzle and is nebulized in small droplets [12]. The atomized droplets are ejected and collected into a tank placed on a rotating platform. Into the collecting tank, FS drops enter in contact with a CaCl_2_ solution and immediately gelate (ionotropic gelation). The spray-gun, the collecting/gelation tank and the rotating platform are placed in a hood to remove the small fraction of atomized FS dispersed in the environment. After gelation, MPs are filtered, washed twice with absolute ethanol and dried overnight at 37 °C in an oven.

### 2.2. Preliminary Experiments for Parameter Setting

Preliminary experiments were performed with the aim of establishing the CaCl_2_ concentration suitable to yield MPs with all -or most- of the FSs of interest (*i.e.*, alginate concentration from 1% w/v to 5% w/v). CaCl_2_ concentrations lower than 5% w/v were not considered suitable since unable to provide a fast droplet gelation and leading to the formation of large aggregates. Among the other concentrations tested (>5% w/v), 15% w/v was recognized as the most versatile, allowing to yield MPs with any of the FSs tested and operational conditions adopted.

A number of eight preliminary batches were also produced in order to identify the range of viscosity of FSs that could be processed and to optimize the production settings (Table 1). A qualitative scoring was attributed to the preliminary batches produced. The lower scores were attributed to the inhomogeneous spraying and/or nozzle clogging, the formation of alginate aggregates in the gelation fluid, and the final product with poor flow properties.

According to the qualitative scoring, the large nozzle was more suitable to nebulize FSs with high alginate concentrations. Viscosity became problematic (too much viscous to be processed) when alginate concentration was increased up to 5% w/v (Table 1). Conversely, the small nozzle was the most suitable to process FSs with alginate concentration of 2% w/v (Table 1).

**Table 1:** Operative settings of the preliminary batches produced.

Batch number	Nozzle’s orifice	Alginate (% w/v)	CaCl_2_ (% w/v)	Qualitative scoring
**1**	Large	2	15	+/−
**2**		3		+
**3**		4		+
**4**		5		−
**5**	Small	1		+
**6**		2		+
**7**		3		+
**8**		4		+/−

### 2.3. Prototype’s Production Yield

The production yield of a set of experiments is reported in Table 2. For each FS volume, the losses were calculated by subtracting the dry weight of the batch to the overall original dry weight of all FS components. With the increase of FS volume, the production losses progressively decreased. The losses were mainly due to the aliquot of FS that remained on the walls of the FS chamber and inside the fluid circuit, to the fraction of atomized droplets dispersed in the environment and to the processes of filtration and recovery. Based on these results, the prototype appears to be convenient and to grant a high yield starting from 2 L of FS (Table 2).

**Table 2:** Production yield as a function of feed solution volume.

Batch n.	Volume (L)	FS dry weight (g)	MP dry weight (g)	Yield (%)
3-1L	1	25.4	12.0	47.2
3-2L	2	50.8	35.5	69.9
3-3L	3	76.2	66.7	87.5
3-4L	4	101.6	91.8	90.4

Prototype throughput has been evaluated using the parameter settings of batch n. 3 with the FS at different alginate concentrations (Table 3). Alginate concentrations of 1, 2, and 3% w/v gave high yields with low variability after single atomization and total FS processing. The increase of concentration to 4% w/v produced a reduced but still acceptable yield that however continues to decrease with concentration increase (Table 3).

**Table 3:** Prototype throughput as a function of alginate concentration. Setting: single nebulization, 1 s; pause between two consecutive nebulization, 4 s; overall duration of assay, 5 min (60 cycles); piston pump inlet pressure, 2 bar; nozzle diameter, 305 µm.

% w/v alginate	1	2	3	4	4.2	4.4
Single atomization (g)	7.31 ± 0.1	7.04 ± 0.4	7.21 ± 0.8	6.68 ± 0.1	6.38 ± 0.1	6.08 ± 0.04
Total (g)	438.2 ± 4.6	422.4 ± 22	432.7 ± 46.2	400.5 ± 21.5	382.7 ± 6.1	366.5 ± 4.4

### 2.4. Rheological Analysis

**Figure 2:**
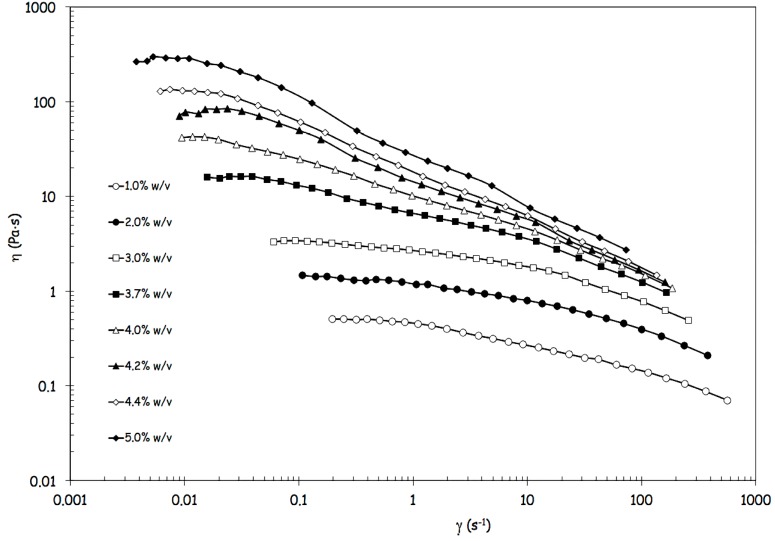
Flow curves of feed solutions containing alginate in a range from 1% w/v to 5% w/v, in terms of viscosity *versus* shear rate.

Since the prototype was designed to process highly viscous FSs and, in preliminary experiments, alginate concentration showed to influence the production yield, a deep rheological characterization was performed on the different FSs. The flow curves of FSs, showed in Figure 2, are reported in terms of viscosity *versus* shear rate. 

Data of Figure 2 clearly show how the shear thinning behavior becomes increasingly evident with increasing alginate concentrations, because the increase of viscosity is more pronounced at the lower shear rates [14]. In parallel, the critical shear rate marking the transition from the upper Newtonian plateau to the shear thinning region shifts at lower values (Figure 2). The flow behavior can be satisfactorily described with the Carreau-Yasuda equation (Equation 1),

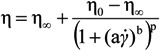
(1)
where the infinite shear viscosity η_∞_ can be arbitrarily set equal to zero in the absence of experimental data at sufficiently high shear conditions. Therefore, the effects of alginate concentration can be synthetically analyzed through the model parameters, in particular, through the variations of the zero shear viscosity η_0_ and the critical shear rate 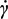
_c_, derived from model parameters (a, b and p) in correspondence with η = η_0/2_.

**Figure 3:**
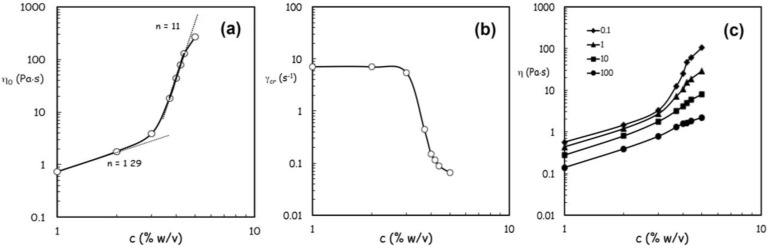
Flow behavior of feed solutions containing alginate in a range from 1% w/v to 5% w/v according to Carreau-Yasuda equation. Panel (**a**): effect of alginate concentration on the variations of the zero shear viscosity η_0_; Panel (**b**): effect of alginate concentration and on the critical shear rate 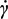
_c_, at infinite shear viscosity η_∞_ set equal to zero; and Panel (**c**): effects produced by an increase of alginate concentration above 3% w/von viscosity values at different reference shear conditions (0.1, 1, 10, 100 s^−1^).

An evident transition occurs in the concentration dependence of both parameters around 3% w/v, as illustrated in Figure 3a,b. At low concentration the rate of increase in zero shear viscosity η_0_ falls in the typical range of dilute solutions while it is much higher at concentrations above 3% w/v. Such a high scaling law exponent, exceeding that of ordinary concentrated polymer solutions, can be attributed to structural interactions between alginate and (hydroxypropyl)methyl cellulose (HPMC) promoted by the co-presence of lysozyme and ethanol [15,16].

The pronounced drop in the critical shear rate *c* occurring above 3% w/v underlines how much the extension of the upper Newtonian plateau is confined at very low shear rates at higher alginate concentrations owing to the remarkable role played by inter-polymeric associative interactions on the rheological properties of concentrated FSs.

By comparing the viscosity values at different reference shear conditions (0.1, 1, 10, 100 s^−1^), it can clearly be noticed that the effects produced by an increase of alginate concentration above 3% becomes more and more remarkable at lower shear rates (Figure 3c).

**Figure 4:**
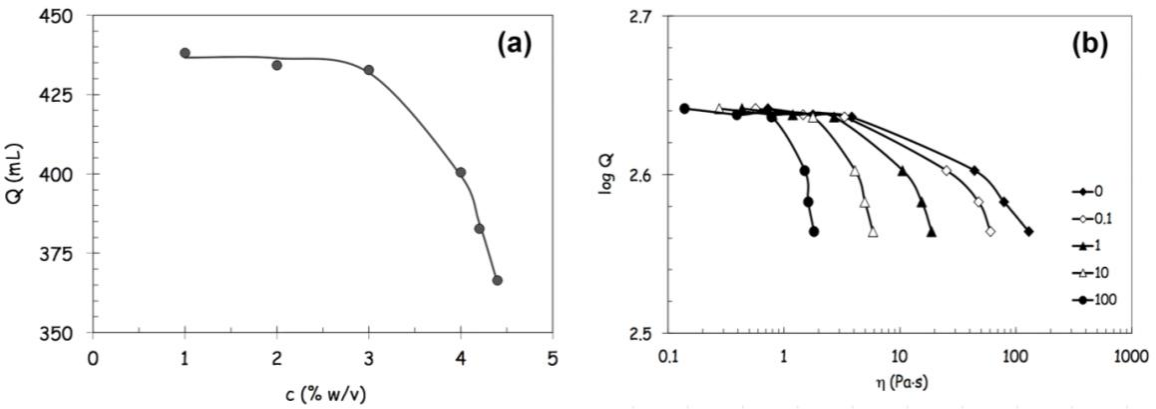
Prototype throughput analysis. Panel (**a**): prototype throughput *Q* as a function of alginate concentration in feed solutions; and Panel (**b**): correlation between throughput and viscosity. The zero-shear viscosity η_0_ and the viscosity values are calculated, at different reference shear rates, from the Carreau-Yasuda equation.

Figure 4a shows that the throughput *Q* begins to decrease more significantly above 3% w/v with a profile that can be described by the following stretched exponential relation (Equation 2):

*Q* = 437 exp (−(0.18 *c*)^7.2^) (2)

where *c* is the alginate concentration.

The correlations between throughput and viscosity (the zero-shear viscosity η_0_ and the viscosity values calculated at different reference shear rates from the Carreau-Yasuda equation, Equation 1) are illustrated in Figure 4b, where the throughput decays again show stretched exponential profiles. The throughput began to decrease more rapidly when the viscosity exceeded a critical threshold value, which depended on the shear rate considered as reference value for flow conditions. Furthermore, the decay increased with increasing shear rate and tended to become even more pronounced at the highest shear rates, tending to approach the process flow conditions encountered within the nozzle. The poor process ability of the most concentrated FS (5% w/v) appears to be better correlated with its higher viscosity values displayed with high shear conditions.

Notwithstanding the satisfactory correlations found between throughput and viscosity values, it must be emphasized that the flow conditions experienced by the fluid in the prototype are intermittent and characterized by significant extensional components within various segments of the process, particularly in sudden contraction regions, and then far from the steady conditions established for the shear viscosity measurement.

A more comprehensive rheological characterization should include transient flow conditions and time-dependent viscoelastic responses as well as extensional viscosity measurements, so providing the experimental data basis necessary for testing the numerical simulation of the macroscopic behavior exhibited in the process configuration. Such a formidable task is outside of the aim of the present work.

### 2.5. Microparticle Characterization

Four of the preliminary batches (n. 1, 3, 6, and 8) were re-prepared in order to characterize the products obtained (Table 4) in terms of particle size (also after swelling), morphology, LZ content and release in different media. Two FSs characterized by a different viscosity (high viscosity, H, and low viscosity, L), corresponding to the previously described “alginate 4% w/v” and “alginate 2% w/v”, with viscosities of about 100 Pa∙s and 1 Pa∙s respectively, were employed. LZ was added to each FS up to a final concentration of 4.7 g/L (Table 4). A 15% w/v CaCl_2_ was used as gelation fluid. Nebulization, gelation and further processing were done as previously described.

**Table 4:** Operative setting for the batches extensively characterized.

Batch n.	% w/w			Viscosity of FSs: (H, high; L, low)	Nozzle size
	Alginate	HPMC	LZ		
**6**	72.8	8.7	18.5	H	Small
**1**	72.8	8.7	18.5		Large
**8**	57.3	13.6	29.1	L	Small
**3**	57.3	13.6	29.1		Large

**Figure 5:**
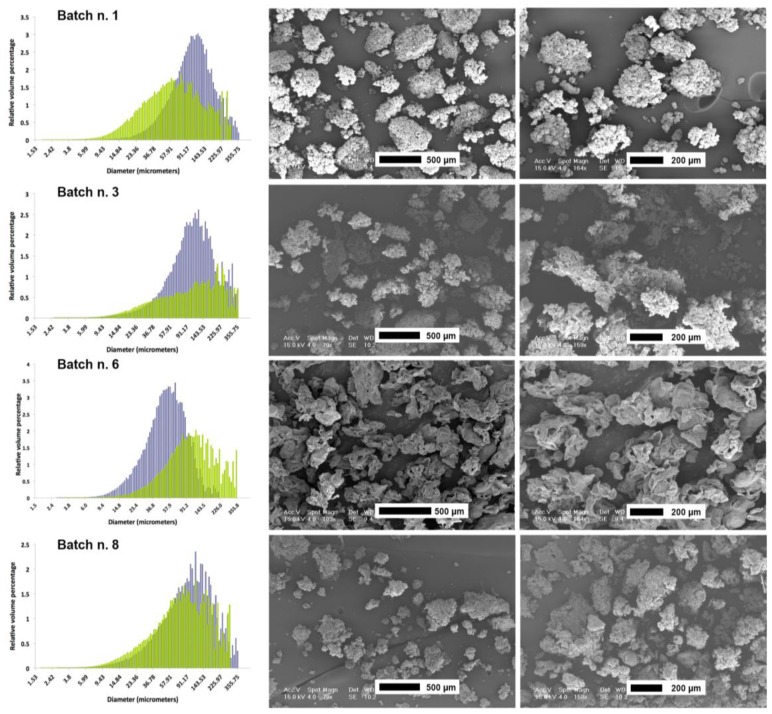
Particle size distribution (bluish, native particle distribution; green, particle distribution after swelling in phosphate buffer saline) and scanning electron microscopy photographs of the four batches produced for characterization.

Mean particle size and distribution have been analyzed on the particle fraction smaller than 400 μm after batch sieving. The fraction with the largest particles (> 400 μm) was very tiny for the batches n. 1, 3, and 6 with 5.0% w/w, 7.1% w/w, and 3.0% w/w, respectively, while the batch n. 8 had a large particle fraction of about 18% w/w. The volume mean diameter (Vmd) and the particle size distribution of the fraction < 400 μm are reported in Table 5 and illustrated in Figure 5, respectively. Batches n. 1 and 6, both prepared with high viscosity solutions but with the small and large size nozzles, respectively, presented a smaller Vmd than batches n. 3 and 8, prepared with low viscosity solutions (Table 5). Even though only the first two batches seemed to have a symmetric Gaussian distribution, particle distributions were similar for all the batches (Figure 5).

Scanning electron microscopy (SEM) pictures, reported in Figure 5, show that particles from batchn. 6 have a morphology different from the others. In fact, these particles were very irregular with crumpled surfaces and a non-spherical shape.

**Figure 6:**
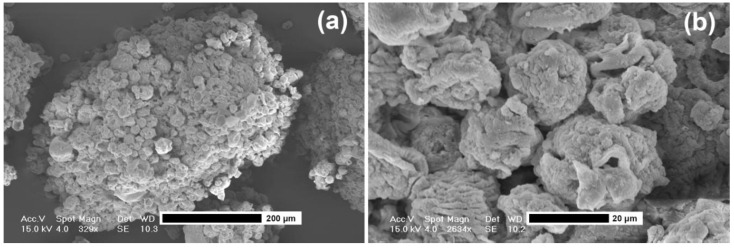
SEM photographs of microparticle surface morphology (batch n. 1). (**a**): Magnification, 329×; and (**b**): magnification, 2634×.

On the contrary, particles of the other batches appear to be formed by the aggregation of smaller spherical particles that probably occurred during the impact with the gelation solution (Figure 5). This observation is of interest to foresee the potentiality of this novel airless spray-gun microencapsulation system. In fact, it can be speculated that there is the possibility to produce particles of a smaller size (20–50 µm) than those presented here (Figure 6). This could be of interest to produce powders with a particle size closer to that generally obtained using spray-drying technology.

**Table 5:** Volume mean diameter of microparticles before and after swelling.

Batch n.	Native Vmd (µm)	After swelling in H_2_O	After swelling in PBS
**1**	158.7 ± 108.8	144.7 ± 105.3	153.6 ± 136.3
**3**	184.5 ± 122.7	189.8 ± 123.5	274.4 ± 131.7
**6**	68.9 ± 61.1	51.5 ± 45.3	137.5 ± 125.5
**8**	190.8 ± 132.2	175.9 ± 125.5	199.0 ± 142.0

Since the airless spray-gun apparatus has been conceived for large scale production of microparticulate systems starting from very viscous and bulky materials, powder characteristics like flowability was investigated. The angles of repose of the four batches investigated are reported in Table 6. Powders from batches n. 1, 3, and 6, had an angle of repose that was found lower than 35° while the powder of the batch n. 8 showed a value slightly higher than 35°. Generally, an angle of repose smaller than 35° indicates a good flowability with no concerns for the industrial processes [17].

**Table 6:** Angle of repose.

Measure	Batchn. 1	Batch n. 3	Batch n. 6	Batch n. 8
1	32.62	32.21	31.80	36.87
2	32.21	32.21	32.21	38.31
3	33.02	33.42	31.38	37.23
Mean ± S.D.	32.6 ± 0.41	32.6 ± 0.70	31.8 ± 0.42	37.5 ± 0.75

Counterintuitively, in this specific case, the larger particle size corresponded to the higher angle of repose. Generally, it is believed that the larger is the particle size, the better is the flowability but this was not the case. Obviously, the particle size and distribution are not the only characteristics that influence the flowability and particle morphology, porosity, and surface area should be taken into account as well. By analyzing the surface morphology at higher magnification, particles seemed to derive from the stable aggregation of smaller particles as previously stated. However, the building blocks (the small particles) are not spherical like those of the batch n. 1 (Figure 6) showing a collapsed and shriveled shape, probably due to their impact with the gelation solution. Even whether these characteristics are shared with batch n. 3, overall particle surface of the batch n. 8 is smoother (data not shown) and this may confer the larger surface for particle-particle interaction, conferring a lower flowability.

Vmd and particle size distributions before and after swelling are reported in Table 5 and illustrated in Figure 5. As expected, all the investigated particles, but those of batch n. 1, swollen after incubation in PBS because of the calcium extraction operated by the phosphate. On the contrary, water was able to swell and increase the Vmd only of the formulation n. 3 (Table 5).

The effect of phosphate on calcium alginate is known and reported in literature [18,19] and it is ascribed to the affinity between phosphate and calcium ions, leading to a de-gelling of the system. In this specific case, the presence of an outer chitosan coating does not allow for fast particle destruction [19]. The effect of water (reduction of Vmd) can be explained in a different way. Generally, calcium alginate MPs, especially when stabilized by a polycation outer layer, are stable in distilled water for months. The peculiar particle structure (agglomerates of smaller particles as shown in Figure 6) might allow the detachment of small particles from the surface, reducing effectively the Vmd of the powder. However, the decrease of the particle optical density [20] leading to minor light obscuration when travelling into the measure cells [21] cannot be excluded. If this was the case, particles even larger (swollen) than the original ones (dry) would have been seen smaller and the recorded Vmd reduction was only apparent.

Finally, MP release behaviors were characterized to assess the possibility to use LZ-embedded MPs as a prolonged delivery system in fish farming. To this aim, release studies were performed at room temperature and not at 37 °C, even though fish body temperature may be lower. In fact, fresh water fishes are poikilothermic animals and their body temperature fluctuates as a consequence of variation in the ambient environmental temperature [22].

**Figure 7:**
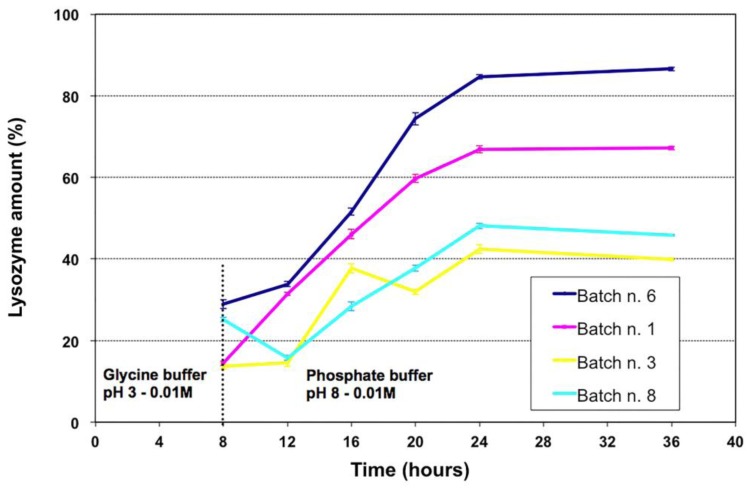
Lysozyme release patterns at pH 3 and at pH 8 of the four selected batches. Microparticles were incubated 8 hours in glycine buffer (pH 3) to mimic gastric environment and, successively, in phosphate buffer saline (pH 8) to mimic small bowel’s environment.

Figure 7 shows LZ release from MPs when incubated 8 hours in glycine buffer (0.01 M, pH 3), to mimic gastric environment, and subsequently in PBS (0.01 M, pH 8). MPs released about 15%–30% of the loaded LZ in the acidic environment with the batches n. 1 and 8 releasing around 15%. The resistance to gastric environment is generally due to the outer chitosan coating and similar behavior has been previously observed with chitosan-coated alginate MPs [23,24].

After removing the acidic buffer and submitting MPs to conditions mimicking intestinal environment, larger differences were observed between the four batches (Figure 7). MPs from the batches n.3 and 8 released less than 50% of their content while batches n. 1 and 6 released ~70% and ~90%, respectively.

Batch n. 6 seems the most promising because of the gradual and almost complete release, even though about 30% of the content was released in the acidic environment. However, the time of acidic incubation has been exacerbated due to the variation of the gastric transit time for different fish species.

## 3. Experimental Section

### 3.1. Materials

Food-grade sodium alginate and food-grade chitosan were purchased from Quingdao Bright Moon Seaweed Group Co., Ltd, Jiaonan, Quingdao, China. Pharmaceutical grade HPMC was obtained from Cigenmann-Veronelli, Milan, Italy, while food grade hen egg white lysozyme, was purchased from Belovo Sa., Bastogne, Belgium. All other chemicals and reagents were of analytical grade.

### 3.2. Instrumentation

Air compressor: model Silent 1, Shamal Compressor, Piossasco, Italy.

Air dryer with custom-made battery of filters: FR Compressori, Trieste, Italy.

Piston Pump: model Merkur, Graco Inc., Minneapolis, MN, USA.

Airless spray-gun: Automatic Airless Spray Gun, Model 288048, Graco Inc., Minneapolis, MN, USA.

Tip with nozzle: model FFT412 with nozzle diameter of 305 µm (hereafter named “large”) and FFT308 with a nozzle diameter of 203 µm (hereafter named “small”) from Graco Inc., Minneapolis, MN, USA.

Custom-made timer: Megaspray, Pordenone, Italy.

Custom-made rotating platform: Delta Instruments, Trieste, Italy.

#### 3.2.1. Prototype Description

A simplified scheme of the airless spray-gun prototype is presented in Figure 1. Description of the single parts is following: (1) stainless still tank that contains the FS; (2) air compressor and air treatment system; (3) spray-gun pressure regulator; (4) piston pump pressure regulator; (5) piston pump for fluids; (6) timer; (7) Graco^®^ spray-gun; (8) spray-gun’s tip with nozzle and tip guard; (9) nebulized drops of FS; (10) rotating gelation and collecting tank; and (11) gelation/coating fluid.

#### 3.2.2. Microparticle Production

FS were prepared according to previously reported formulations [4]. The previous study demonstrated that the viscosity of the FS is directly proportional to the concentration of the food grade alginate employed, with minor influence due to other components. In order to identify the operational limits of the prototype, due to the viscosity of the fluids by varying the percentage of alginate, FSs with different viscosities were prepared. Briefly, to prepare 1 L of FS we firstly dissolved an “x” amount of alginate in 453 mL of distilled water. Subsequently, 4.7 g of LZ and 547 mL of a solution of HPMC were added. HPMC solution was prepared by dissolving 10 g of HPMC in 1 L of ethanol absolute and, then, by adding 1.5 L of distilled water). The “x” values were chosen to obtain a final alginate concentration of: 1, 2, 3, 4 and 5% w/v.

The gelation/coating fluid was prepared as follows: 1 g of chitosan was added to 1 L of water containing 1.25 mL of acetic acid and CaCl_2_ was added as ionotropic gelation agent to achieve different final concentrations.

In order to assess the production yield in relation to the FS’s volume, four different volumes of FS were nebulized: 1, 2, 3 and 4 L. For each batch, the production yield was calculated with the following formula:

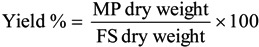
(3)


#### 3.2.3. Operative Conditions

Operative pressures values—Air compressor’s air pressure outlet, 7 bar; spray-gun’s air pressure inlet, 2.8 bar; piston pump’s air pressure inlet, 2 bar; piston pump’s FS outlet, 80–100 bar (value estimated on the basis of product specification; the value can be influenced by the viscosity of the FS).

Spraying settings—The nebulization single impulse time length was of 1 s. The time length of the pause between two consecutive nebulization impulses was of 2 s.

Nozzles—Two nozzles, which differ for the orifice of different caliber and henceforth indicated as “small” and “large”, were assayed for batch production.

Gelation fluids—In order to identify the optimal concentration of gelation fluid, three different concentrations of CaCl_2_ were employed: 3% w/v, 5% w/v and 15% w/v.

### 3.3. Rheology

Rheological tests were performed under continuous shear conditions to determine steady viscosity values in the stress range 0.01–1000 Pa, as well as under oscillatory shear conditions to determine the extension of the linear viscoelasticity regime (stress sweep tests at 1 Hz) and to determine the mechanical spectrum (frequency sweep in the range 0.01–50 Hz).

All tests were carried out at 25 °C using the controlled stress rheometer Rheostress Haake RS 150 equipped with a cone/plate measuring device (diameter: 60 mm, angle 1°) and, at higher polymer concentrations, with parallel plates with serrated titanium surfaces (diameter: 35 mm, gap: 2 mm). A glass bell covering the measuring device and operating under saturation conditions was used to improve thermal control and eliminate evaporation effects.

A set of FSs at different alginate concentration were prepared for rheological analysis, according to the procedure previously reported. The alginate concentrations of the samples were 1% w/v, 2% w/v, 3% w/v, 3.7% w/v, 4% w/v, 4.2% w/v, 4.4% w/v and 5% w/v, respectively.

### 3.4. Microparticle Preparation and Characterization

A set of four batches was prepared in order to characterize the MPs produced by the prototype. A 15% w/v CaCl_2_ was used as gelation fluid. Nebulization, gelation and further processing were performed as previously described.

Size distribution of the four batches was analyzed by means of an AccuSizer 780/AD Autodiluter equipped with an optical particle counting unit [13]. Before analysis, the 4 powders underwent sieving with a 400 micrometers sieve in order to quantify the mass of particles larger than 400 µm and to avoid instrument blockage. A sample of particles for each sieved batch was dispersed in ultrapure water under vortexing and analyzed immediately. A second set of samples was dispersed in ultrapure water while a third in phosphate buffer saline (PBS) (0.1 M, pH 8). Both samples were left to swell for 24 hours at room temperature and then analyzed.

MP shape and surface morphology were investigated by SEM (Philips XL30 SEM, Heindoven, the Netherlands). Samples were prepared by placing the powder onto an aluminum specimen stub covered with a double sided adhesive carbon disc. Mounted samples were sputter coated with gold at 20 mA for 4 min prior to imaging (EMITECH K-550X sputter coater, Ashford, UK).

The angle of repose was determined according to one of the European Pharmacopoeia compendial methods. Briefly, an adequate amount of powder was allowed to flow through a funnel on a Teflon^®^ circular base (diameter 4 cm) until when the formed cone of powder covered the entire Teflon^®^ base. Preliminary experiments were performed to fix the end of the funnel at about 4 cm from the tip of the powder cone [17]. The height of the cone was measured with a laser ray parallel to the plate surface and moving up and down perpendicularly to the plate surface. The angle of repose was calculated from tangent α, using the following equation:

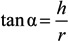
(4)
were *h* is the measured height of the cone; and *r* is the radius of the plate (2 cm).

LZ content in the MPs and the amount released were determined by the microBCA (bicinchoninic acid) total protein assay method [25]. Calibration curve was built analyzing in triplicate five different concentrations of LZ solution from the same batch employed for encapsulation. Preliminary investigations were performed to rule out the possible interference of the single MP components on microBCA assay released during particle destruction. Since the components did not interfere with the analysis at the concentrations theoretically present, MPs were destroyed by adding PBS (0.1 M, pH 8) and treating at 37 °C with ultrasounds. Blank assays with the sole LZ were performed to rule out the possibility of peptide degradation during MP destruction. After the aforementioned procedure, samples were centrifuged and the supernatant was withdrawn and analyzed to evaluate LZ concentration by microBCA assay. Measurements were performed in triplicate and the error was expressed as standard deviation.

Release studies have been performed in two steps. The four batches have been first incubated in glycine buffer (0.l M, pH 3) at room temperature and the total amount of LZ released over eight hours was quantified using microBCA assay, as previously described. After eight hours in glycine buffer, particles were centrifuged, the supernatant was completely removed and substituted with PBS (0.1 M, pH 8). The release was then followed for 24 hours at room temperature.

## 4. Conclusions

The new prototype for microencapsulation of bioactive principles, here described, has the main characteristic of processing large amounts of highly viscous FSs. Unlike the currently available technologies at the same scale and cost, this prototype easily processes, in a few hours, several liters of FSs in a range of η from 0.5 up to more than 100 Pa∙s. Due to the not irrelevant death volume in the fluid circuit, the plant, as currently engineered, is not particularly adapt for small scale laboratory productions but it is suitable for pre-industrial scale productions e.g. field trials (in aquaculture or for other animal husbandry applications) or large preclinical and clinical trials. The throughput of the prototype was linear up to 3% w/v of alginate concentration with a slight modification only in case of FSs with higher degree of viscosity. At the maximum degree of viscosity tested the throughput decrease was 15% w/v. The particles size distribution and the flow properties of the batches produced are considered satisfactory. The aspect of the particles show highly corrugated surface. The release of lysozyme, used as model bioactive principle, was low at low pH while occurring mostly at the pH simulating the intestinal environment, showing gastro-resistance and suitability for oral applications. The overall characteristics of the batches produced are viscosity- and prototype settings-sensitive. The aggregates of MPs obtained in some of the batches suggest the need of a careful setting of the operative conditions in order to avoid this phenomena and increase the reproducibility.

The highest percentage of bioactive principle successfully included in MPs with the here presented prototype is 40% w/w, up to date (data not shown). These MPs are object of an on-going research aimed to the oral delivery of hen egg white LZ on farmed trout. For what the cost effectiveness of the processing is concerned, we can highlight that is particularly interesting up to the semi-finished product (wet gelified MPs). As expected, the main cost of the procedure is concentrated on the last step: particle recovery and drying.

In conclusion, the prototype appears to be suitable for pre-industrial scale production of microparticulate systems by processing FSs in a wide range of viscosities.
